# Epidemiological and clinical implications of asymptomatic malaria and schistosomiasis co-infections in a rural community in western Kenya

**DOI:** 10.1186/s12879-021-06626-2

**Published:** 2021-09-09

**Authors:** Edwin Kamau, Adam Yates, Risper Maisiba, Valentine Singoei, Benjamin Opot, Rose Adeny, Cornel O. Arima, Victor Otieno, Catherine S. Sumbi, Raphael O. Okoth, Farid Abdi, Maurine Mwalo, Jew Ochola, June Otieno, Julie Ake, Michelle Imbach, Hannah A. Turley, Dennis Juma, Hoseah M. Akala, John Owuoth, Ben Andagalu, Trevor A. Crowell, Chiaka Nwoga, Jessica Cowden, Christina S. Polyak, for the RV393 Study Group, Rachel Adongo, Rachel Aguttu, Michael Bondo, Erica Broach, Christine Busisa, Nate Copeland, Mark de Souza, Leigh Anne Eller, Milicent Gogo, Zebiba Hassen, Dale Hu, Anne Juma, Oscar Kasera, Qun Li, Margaret Mbuchi, Mark Milazzo, Kayvon Modjarrad, Eric Ngonda, Jacob Nyariro, Roseline Ohore, Thomas Okumu, Mary Omondi, Cephas A. Oyieke, Everlyne E. Omondi, Vincent L. Akolo, Agneta A. Ogolo, Michael O. Ayaya, Timothy Omondi, Linnah Ooro, Beatrice Orando, Victorine Owira, Roselyn Oyugi, Merlin Robb, Eric Rono, Chi Tran

**Affiliations:** 1 United States Army Medical Research Directorate-Africa, Nairobi, Kenya; 2 HJF Medical Research International, Kisumu, Kenya; 3 U.S. Military HIV Research Program, Walter Reed Army Institute of Research, Silver Spring, MD USA; 4 Henry M. Jackson Foundation for the Advancement of Military Medicine, 6720A Rockledge Drive, Suite 400, Bethesda, MD 20817 USA; 5 Kenya Medical Research Institute, Kisumu, Kenya

**Keywords:** *Plasmodium falciparum*, Asymptomatic Malaria, Schistosomiasis, Hematological, Thrombocytopenia, Eosinophilia, Creatinine, Kenya

## Abstract

**Background:**

Malaria and schistosomiasis present considerable disease burden in tropical and sub-tropical areas and severity is worsened by co-infections in areas where both diseases are endemic. Although pathogenesis of these infections separately is well studied, there is limited information on the pathogenic disease mechanisms and clinical disease outcomes in co-infections. In this study, we investigated the prevalence of malaria and schistosomiasis co-infections, and the hematologic and blood chemistry abnormalities in asymptomatic adults in a rural fishing community in western Kenya.

**Methods:**

This sub-study used samples and data collected at enrollment from a prospective observational cohort study (RV393) conducted in Kisumu County, Kenya. The presence of malaria parasites was determined using microscopy and real-time-PCR, and schistosomiasis infection by urine antigen analysis (CCA). Hematological analysis and blood chemistries were performed using standard methods. Statistical analyses were performed to compare demographic and infection data distribution, and hematologic and blood chemistry parameters based on different groups of infection categories. Clinically relevant hematologic conditions were analyzed using general linear and multivariable Poisson regression models.

**Results:**

From February 2017 to May 2018, we enrolled 671 participants. The prevalence of asymptomatic *Plasmodium falciparum* was 28.2% (157/556) and schistosomiasis 41.2% (229/562), with 18.0% (100/556) of participants co-infected. When we analyzed hematological parameters using Wilcoxon rank sum test to evaluate median (IQR) distribution based on malarial parasites and/or schistosomiasis infection status, there were significant differences in platelet counts (p = 0.0002), percent neutrophils, monocytes, eosinophils, and basophils (p < 0.0001 each). Amongst clinically relevant hematological abnormalities, eosinophilia was the most prevalent at 20.6% (116/562), whereas thrombocytopenia was the least prevalent at 4.3% (24/562). In univariate model, Chi-Square test performed for independence between participant distribution in different malaria parasitemia/schistosomiasis infection categories within each clinical hematological condition revealed significant differences for thrombocytopenia and eosinophilia (p = 0.006 and p < 0.0001, respectively), which was confirmed in multivariable models. Analysis of the pairwise mean differences of liver enzyme (ALT) and kidney function (Creatinine Clearance) indicated the presence of significant differences in ALT across the infection groups (parasite + /CCA + vs all other groups p < .003), but no differences in mean Creatinine Clearance across the infection groups.

**Conclusions:**

Our study demonstrates the high burden of asymptomatic malaria parasitemia and schistosomiasis infection in this rural population in Western Kenya. Asymptomatic infection with malaria or schistosomiasis was associated with laboratory abnormalities including neutropenia, leukopenia and thrombocytopenia. These abnormalities could be erroneously attributed to other diseases processes during evaluation of diseases processes. Therefore, evaluating for co-infections is key when assessing individuals with laboratory abnormalities. Additionally, asymptomatic infection needs to be considered in control and elimination programs given high prevalence documented here.

**Supplementary Information:**

The online version contains supplementary material available at 10.1186/s12879-021-06626-2.

## Background

Malaria and schistosomiasis are the two most prevalent parasitic infections in tropical and sub-tropical regions, accounting for a considerable proportion of global morbidity and mortality. Over 90% of these infections occur in sub-Saharan Africa (sSA) with large geographical overlap, making co-infection common [1–4]. Social, economic, and environmental factors are important determinants in the patterns and prevalence of malaria and schistosomiasis including co-infections, with poor and rural communities highly impacted. Further, individuals who engage in certain activities and occupations such as anglers are at increased risked due to increased environmental exposures [1, 4].

*Plasmodium falciparum* accounts for more than 99% of malaria cases in sSA [1]. These infections cause a spectrum of clinical illness ranging from asymptomatic to severe disease with immune status acting as a critical determinant of disease progression [5, 6], and they can be either microscopic or submicroscopic [7, 8]. Malaria infection can lead to hematologic abnormalities such as anemia, leucopenia, and thrombocytopenia, which are important in disease pathophysiology. After repeated exposure, premunition may lead to persistent, asymptomatic infections [5, 9], which are characterized as subclinical due to lack of overt clinical symptoms [6, 8]. However, it is now evident that asymptomatic infections may cause harm to the individual [6, 10], and are reservoirs for transmission [8].

*Schistosoma mansoni* and *S. haematobium* are responsible for the majority of schistosomiasis infections in sSA [4]. These infections can lead to several acute and chronic illness profiles, which may result in growth stunting, cognitive impairment, and death [11]. Schistosomiasis leads to hematologic abnormalities including eosinophilia and leukocytosis. In patients with hepatosplenic disease, anemia is common, and significant increases in monocytes, lymphocytes, and neutrophils have been reported [12], as have mild eosinophilia and neutropenia [13].

While the pathogenesis of *P. falciparum* and *Schistosoma* as separate infections are well studied, pathogenic disease mechanisms and clinical disease outcomes during co-infections are not clear [14]. Some studies have shown increased odds of *P. falciparum* infections and/or malaria-related complications associated with *Schistosoma* co-infections [3, 15, 16] while others have shown no such association [17, 18] or lower incidence of *P. falciparum* infection among children with *S. haematobium* infection [19, 20]. A systematic review of 12 studies based on 9,337 children in eight sSA countries revealed co-infection with *Schistosoma* may increase vulnerability of children to asymptomatic *P. falciparum* infection [14], suggesting chronic infection with *Schistosoma* may worsen clinical outcomes of asymptomatic *P. falciparum* infections.

Both malaria and schistosomiasis are holo-endemic in the fishing communities bordering Lake Victoria in rural western Kenya. In this region, the prevalence of *P. falciparum* infections in children is over 40% [21], and *S. mansoni* is the most prevalent schistosomiasis infection, with a recent study reporting prevalence > 90% in children using a point-of-care test for urinary circulating cathodic antigen (POC-CCA) [22]. Further, in a seroprevalence study, the majority of *S. mansoni* infections (94%) were present in communities < 1.5 km from the lake [23]. Malarial and schistosomiasis infection studies have mostly focused in children because they are more vulnerable and likely to be symptomatic. However, the adult population play an important role as reservoir in the transmission of both infections, and there may be important health implications of chronic infections.

In order to further an understanding of the pathogenesis of *P. falciparum* and schistosomiasis co-infections in adults, this study first characterizes the prevalence of co-infections in asymptomatic adult population in a fishing community bordering Lake Victoria in rural western Kenya. We then examine the association of hematologic and blood chemistry parameters with co-infection status.

## Methods

### Study area and population

Samples and data used in this study were obtained from participants enrolled in a prospective observational cohort study to determine HIV incidence and assess the site’s suitability for future HIV-prevention trials in Kombewa, Kisumu County, Kenya (RV393). Briefly, to be eligible for the study, individuals had to be without HIV, aged 18–35 years, reported two or more sexual partners in the last three months, and willing to reside in Kisumu County for the entire 24-month duration of the study. Participants underwent medical history-taking, physical examination and tests for HIV, malaria, syphilis, schistosomiasis, and hepatitis B and C at enrollment. Samples and data used in this analysis included all study participants with malaria and schistosomiasis diagnostic testing results available from enrollment between February 2017 and May 2018.

### Laboratory testing

Venipuncture was performed for collection of whole blood and serum using Vacutainer® blood collection tubes (Beckton Dickinson, Plymouth, United Kingdom). Hematology parameters were determined from whole blood using a Coulter Ac•T™ 5diff CP analyzer (Beckman Coulter, France). The tests were performed within 24 h of sample collection per manufacturer’s instructions. To test for liver and kidney functions, blood chemistries were performed to test for alanine aminotransferase (ALT) and creatinine. Blood was collected and allowed to clot for a minimum of 30 min, centrifuged, and serum was analyzed on the same day of separation. ALT and creatinine tests were analyzed using the Cobas® c311 biochemistry analyzer (Roche, Germany) according to the manufacturer’s instructions.

Malaria microscopy (smear) was performed following WHO standard method, and as previously described [24]. Briefly, giemsa-stained films were prepared and read by two independent expert microscopists. The findings of two (or three in case of discrepancies) independent expert microscopists were considered. For the detection of *Plasmodium* by molecular diagnosis, real-time PCR was performed as previously described [25]. Briefly, genomic DNA was extracted using the QIAamp DNA mini kit (Qiagen, CA, USA) as recommended by the manufacturer. PCR targeting *Plasmodium* genus- or species-specific 18 s rRNA genes was performed. The appropriated controls were included in every run. Qualitative data was used for PCR result analysis where cycle threshold (Ct) values < 40 was considered positive.

Urine samples collected at enrollment were tested using a commercially available POC-CCA test (Rapid Medical Diagnostics, Pretoria, South Africa) for detection of *S. mansoni* and *S. haematobium* antigens per manufacturer’s recommendation.

### Statistical analyses

#### Data processing/variable formation

Self-identified biologic sex was recorded as ‘Male’ and ‘Female’. Age was categorized as 18–24, 25–29, and 30+ . Marital status was dichotomized into “Married” (married monogamous, married polygamous, cohabitating (come we stay)) and “Not Married” (single, separated, divorced, widowed, other). Self-identified literacy category was recorded as “Yes” or “No” when respondents were asked “Can study participant read and write?”. Educational level was categories into three groups, “None or Some Primary”, “Primary or Some Secondary”, and “Secondary or above” and employment was dichotomized into “Yes” and “No”.

Body mass index (BMI) was calculated using the standard formula, Weight (kg)/height (m)^2^, and BMI was grouped based on international categorical definitions: underweight = BMI < 18.5, normal weight = 18.5 <  = BMI <  = 24.9, overweight/obese: BMI >  = 25. We combined overweight/obese categories due to low N for each category alone. Participants were considered as having anemia if their hemoglobin (HGB) levels were below 11.0 g/dL. Creatinine Clearance (CrCL) was calculated based on the Cockcroft-Gault formula, ([140-age] × weight in kg)/(serum creatinine × 72) and was adjusted for women by 0.85.

#### Comparison of malaria infection status by various assays

Three categories of malaria infection status (smear negative [−]/PCR negative [−]; smear negative [−]/PCR positive [ +]; smear positive [ +]/PCR positive [ +]) were created based on the results from the microscopy and PCR tests. Submicroscopic malaria parasitemia was defined as smear negative but PCR positive test. Hematology and chemistry parameters were summarized as medians and interquartile ranges (IQR) and comparisons made using the Wilcoxon rank sum test.

#### Analysis of malaria parasitemia and schistosomiasis groups

A positive *Plasmodium* PCR result was considered indicative of malaria parasitemia (referred in the manuscript as parasitemia or parasite) and a positive urine POC-CCA test was considered indicative of schistosomiasis. Chi-squared Test of independence (for categorical variables) and Wilcoxon rank sum (for continuous parameters) were conducted to examine the statistical association between various hematologic and blood chemistry metrics and infection category.

#### Analysis of association between hematological conditions and co-infection category

Continuous parameters were recategorized into clinically relevant hematologic conditions: thrombocytopenia (platelet count ≤ 150 × 10^3^/µL), neutropenia (Neutrophil count < 1.5 × 10^3^/µL), eosinophilia (Eosinophil count ≥ 0.5 × 10^3^/µL), and leucopenia (white blood cell count ≤ 4.0 × 10^3^/µL). Fisher’s Exact test was used to examine independence between co-infection groups and disease status within the four hematologic conditions.

We constructed a general linear model with the various hematologic parameters as outcomes and infection status as the categorical predictor. Pairwise differences between infection groups were also assessed using the Least-Squared-Means procedure with Tukey Adjustments using the Dwass, Steel Critchlow-Flinger (DSCF) method to allow for comparison between 3 or more groups (Additional file 1).

Lastly, multivariable Poisson regression models with generalized estimating equations (GEE) and robust variance estimators were used to evaluate associations between infection status and each hematologic condition [26, 27]. Age and sex were included in all models as controls for potential confounding from differential live-course effects. 

## Results

Of the 671 participants enrolled in the study, data from 562 individuals collected at enrollment that had complete parasitemia (smear and PCR), and POC-CCA (referred to as CCA) test results were used in the analyses. Table 1 shows demographic and population characteristics for parasitemia infections based on smears and PCR data, grouped into three infection categories; 6 participants were excluded from these analyses as they were smear + /PCR− and considered false positives, reducing the table n to 556. Overall, parasitemia prevalence was 11.9% (66/556) by smears and 28.2% (157/556) by PCR with 16.4% (91/556) of the study participants having submicroscopic parasite infections. There were significant differences in the infection rates based on gender, marriage, education and body mass index.

**Table 1:** Demographic and population characteristics based on smear and PCR results

	Smear−/PCR− n (%)	Smear−/PCR + n (%)	Smear + /PCR + n (%)	p-value
	399 (73.0)	91 (16.7)	66 (11.9)	
*Gender*				** < 0.0001**
Male	181 (61.6)	58 (19.7)	55 (18.7)	
Female	218 (84.5)	33 (12.8)	11 (4.2)	
*Age (years)*				0.76
18–24	217 (72.8)	45 (15.1)	28 (12.1)	
25–29	109 (70.3)	30 (19.4)	16 (10.3)	
30 +	73 (70.9)	16 (15.5)	14 (13.6)	
*Marital status*				**0.009**
Not married	327 (74.8)	64 (14.6)	46 (10.5)	
Married	72 (60.5)	27 (22.7)	20 (16.8)	
*Education*				**0.0005**
None or primary school	47 (56.6)	19 (22.9)	17 (20.5)	
Primary school/some secondary	149 (69.3)	34 (15.8)	32 (14.9)	
Secondary school and above	203 (79.6)	38 (14.9)	14 (5.5)	
*Employed*				0.46
No	11 (91.7)	1 (8.3)	0 (0.0)	
Yes	388 (71.3)	90 (16.5)	66 (12.1)	
*Body mass index*				**0.0003**
Underweight (< 18.5)	23 (69.7)	4 (12.1)	6 (18.2)	
Normal (18.5–24.9)	251 (66.6)	72 (19.1)	54 (14.3)	
Overweight/obese (≥ 25)	125 (85.6)	15 (10.3)	6 (4.1)	

We then analyzed hematologic parameters and the blood chemistry data by comparing differences in the median (IQR) based on parasitemia status, grouped into the three infection categories. Table 2 shows there was significant difference in platelet counts, percent monocytes, eosinophils and basophil. *Plasmodium* infection was associated with lower platelet counts but higher percent monocytes and eosinophil. It is notable that for the hematologic parameters with significant difference among the three infection categories, individuals with submicroscopic parasitemia (smear−/PCR +) had values that were similar to those that were microscopic. Blood chemistry analysis revealed there were significant differences in ALT values across the three infection categories, with submicroscopic and microscopic infections presenting higher median values compared to uninfected (Table 2).

**Table 2:** Median and interquartile ranges for hematologic and chemistry parameters stratified by smear and PCR results

	Smear−/PCR− Median (IQR)	Smear-/PCR + Median (IQR)	Smear + /PCR + Median (IQR)	p-value
*Hematologic parameters*				
	*n = 399*	*n = 91*	*n = 66*	
Hemoglobin (g/dL)	14.3 (13.2,16.5)	14.6 (13.3,16.0)	14.5 (13.7,15.6)	0.4
WBC count (10^3^/µL)	5.5 (4.6,6.5)	5.5 (4.8,6.5)	5.2 (4.3,6.3)	0.16
MCV (fL)	83 (79,88)	82 (78,88)	83.5 (78,88)	0.8
Platelet count (10^3^/µL)	258 (218,301)	238 (207,275)	232 (176,273)	**0.0001**
Neutrophil %	46.8 (40,53.7)	46.6 (37.9,52.7)	43.7 (37,50.1)	0.1
Lymphocyte %	43.5 (37.9,48.7)	40.9 (36.7,48.1)	43.0 (36.2,48.6)	0.6
Monocyte %	3.1 (2.5,4.1)	3.5 (2.6,4.8)	3.6 (2.7,5.6)	**0.004**
Eosinophil %	3.9 (2.0,7.2)	5.2 (2.1,8.9)	5.2 (2.9,9.6)	**0.007**
Basophil %	0.6 (0.5,0.7)	0.6 (0.5,0.7)	0.6 (0.5,0.8)	**0.04**
*Chemistry parameters*				
	*n = 279*	*n = 67*	*n = 60*	
ALT/SGPT (U/L)	15.5 (12.4, 20.2)	19.6 (12.8,26.7)	18.5 (14.9,24.4)	**0.002**
Creatinine Clearance mL/min)	122.5 (106.6–139.2)	117.2 (100.1–134.7)	114.7 (104.2–135.7)	0.3

*WBC*  white blood count, *MCV*  mean corpuscular volume, *ALT*  alanine aminotransferase, *SGPT*  serum glutamate pyruvate transaminase, *IQR*  interquartile range

Of the 562 study participants with complete data, 229 (41.2%) individuals had reactive schistosomiasis (CCA) test results. The proportion of individuals with CCA positive test results was higher in males (52.7% [157/298]) compared to females (27.3% [72/264]). Of the individuals with reactive CCA test results, 43.7% (100/229) were parasitemic (microscopic and submicroscopic), with co-infection significantly higher in males (p < 0.0001) compared to females. The point prevalence for *Plasmodium*/schistosomiasis co-infection in the study population was 18.0% (100/556).

For further analyses, data was organized into four infection categories based on parasitemia and CCA infection groups (parasitemia−/CCA−; parasitemia + /CCA−; parasitemia−/CCA + ; and parasitemia + /CCA +). Table 3 shows demographic and population characteristics based on these four infection categories. The prevalence of co-infections was significantly different based on gender, marriage, education, and BMI categories. Of note, there was no significant difference among the different infection group categories based on anemia, which was excluded from further analysis due to small counts.

**Table 3:** Demographic and population characteristics based on infection group

	Parasite−/CCA−	Parasite + /CCA−	Parasite−/CCA +	Parasite + /CCA +	p-value
	n (%)	n (%)	n (%)	n (%)	
*Gender*					
Male	113 (38)	28 (9)	72 (24)	85 (29)	** < 0.0001**
Female	163 (62)	29 (11)	57 (22)	15 (6)	
*Age (years)*					
18–24	149 (50)	36 (12)	71 (24)	45 (15)	0.32
25–29	81 (51)	13 (8)	31 (20)	33 (21)	
30 +	46 (45)	8 (8)	27 (26)	22 (21)	
*Marital status*					
Not married	247 (56)	49 (11)	84 (19)	61 (14)	** < 0.0001**
Married	29 (24)	8 (7)	45 (37)	39 (32)	
Can read and write*					
No	1 (20)	0	1 (20)	3 (60)	0.12
Yes	275 (49)	57 (10)	128 (23)	97 (17)	
*Education Category*					
None or some primary	23 (27)	6 (7)	25 (30)	30 (36)	** < 0.0001**
Primary or some secondary	97 (45)	21 (10)	53 (25)	45 (21)	
Secondary and above	156 (60)	30 (11)	51 (19)	25 (10)	
Employment status*					
Not employed	6 (50)	0	5 (42)	1 (8)	0.39
Employed	270 (49)	57 (9)	124 (23)	99 (18)	
*Body mass index*					
Underweight (< 18.5)	20 (61)	5 (15)	3 (9)	5 (15)	** < 0.0001**
Normal (18.5–24.9)	167 (43)	36 (9)	92 (24)	90 (24)	
Overweight/obese (≥ 25)	92 (63)	16 (11)	34 (23)	5 (3)	
Anemia (Hb < 11.0 g/dL)*					
No	265 (49)	56 (10)	124 (23)	95 (18)	0.85
Yes	11 (50)	1 (5)	5 (23)	5 (13)	

*Asterisks indicates the use of Fisher’s exact test rather than Chi-Square due to small expected values

We then analyzed hematological parameters by comparing categorical differences in the median (IQR) test results amongst the four infection group categories. There were significant differences in platelet counts, percent neutrophils, monocytes, eosinophils, and basophils (Table 4). Compared to the malaria and schistosomiasis uninfected group, co-infections were associated with lower platelet counts and percent neutrophils, but higher percent monocytes, eosinophils, and basophils. Infection with *Plasmodium* only was associated with reduction in platelet count whereas infection with schistosomiasis only was associated with a large increase in platelet counts and percent eosinophils. Analyses of the blood chemistry revealed there was significant difference in ALT, with the co-infected group category having higher median values compared to uninfected group category. It is notable that infection with *Plasmodium* did not lead to significant change in ALT values compared to those uninfected.

**Table 4:** Median and interquartile ranges for hematologic and chemistry parameters stratified by infection group

	Parasite−/CCA− Median (IQR)	Parasite + /CCA− Median (IQR)	Parasite−/CCA + Median (IQR)	Parasite + /CCA + Median (IQR)	p-value
*Hematologic parameters*					
	*n = 276(49.1)*	*n = 57 (10.1)*	*n = 129 (23.0)*	*n = 100 (17.8)*	
Hemoglobin (g/dL)	14.1 (13.2,15.5)	14.3 (13.5,15.4)	14.8 (13.6,15.7)	14.9 (13.7,16.0)	0.06
WBC count (10^3^/µL)	5.5 (4.6,6.4)	5.3 (4.6,6.5)	5.6 (4.6,6.9)	5.4 (4.6,6.4)	0.74
MCV (fL)	83 (79,87)	84 (79,88)	84 (79,88)	82 (77,87)	0.58
Platelet count (10^3^/µL)	256 (220,303)	242 (212,278)	261 (207,293)	226.5 (199,273)	**0.0002**
Neutrophil %	48.2 (42,54)	47.9 (40.7,54.6)	42.8 (36.6,50.6)	44.1 (35.9, 50.8)	** < 0.0001**
Lymphocyte %	43.7 (38,49)	45.1 (37.6,50.4)	43.3 (38,48.2)	41.3 (35.6,47.7)	0.15
Monocyte %	3.1 (2.5,4)	3.2 (2.4,4.2)	3.4 (2.5,4.6)	4.0 (2.9,6.1)	** < 0.0001**
Eosinophil %	2.8 (1.7,5.5)	2.5 (1.7,5.1)	6.8 (3.9,11.4)	7.1 (4.1, 10.7)	** < 0.0001**
Basophil %	0.6 (0.5,0.7)	0.5 (0.5,0.7	0.6 (0.5,0.8)	0.7 (0.5,0.8)	** < 0.0001**
*Chemistry parameters*					
	*n = 184 (45.7%)*	*n = 35 (7.6%)*	*n = 101 (25.3%)*	*n = 92 (21.4%)*	
ALT/SGPT (U/L)	14.7 (11.7,19.8)	14.1 (11.3,18.1)	17.2 (13.6,21.8)	20.4 (16.2,26.8)	** < 0.0001**
Creatinine clearance (mL/min)	123.0 (107.7–143.5)	113.1 (97.7–133.0)	120.8 (106.6–134.2)	119.1 (106.2–136.0)	0.08

*WBC*  white blood count, *MCV*  mean corpuscular volume, *ALT*  alanine aminotransferase, *SGPT*  serum glutamate pyruvate transaminase, *IQR*  interquartile range

We then performed pairwise comparisons for the continuous hematology variables by parasite/CCA combination using raw cell counts, focusing on hematological parameters of clinical significance including platelet, neutrophil, eosinophil, and WBC counts. This analysis was performed in a non-parametric framework using the DSCF method, which showed parasitemia−/CCA− vs. parasitemia + /CCA + categories were significantly different from each other in platelet and eosinophil counts (p < 0.0001 for each), and parasitemia−/CCA + vs. parasitemia + /CCA + group categories in platelet counts (p = 0.05). Further, comparison of parasitemia−/CCA− vs. parasitemia−/CCA + ; parasitemia + /CCA− vs. parasitemia−/CCA + ; and parasitemia + /CCA− vs. parasitemia + /CCA + group categories in eosinophil counts revealed that they were significantly different (p < 0.0001 for each). There were no significant differences when neutrophil or WBC median values were compared for any of the parasitemia/CCA combinations.

**Fig. 1:**
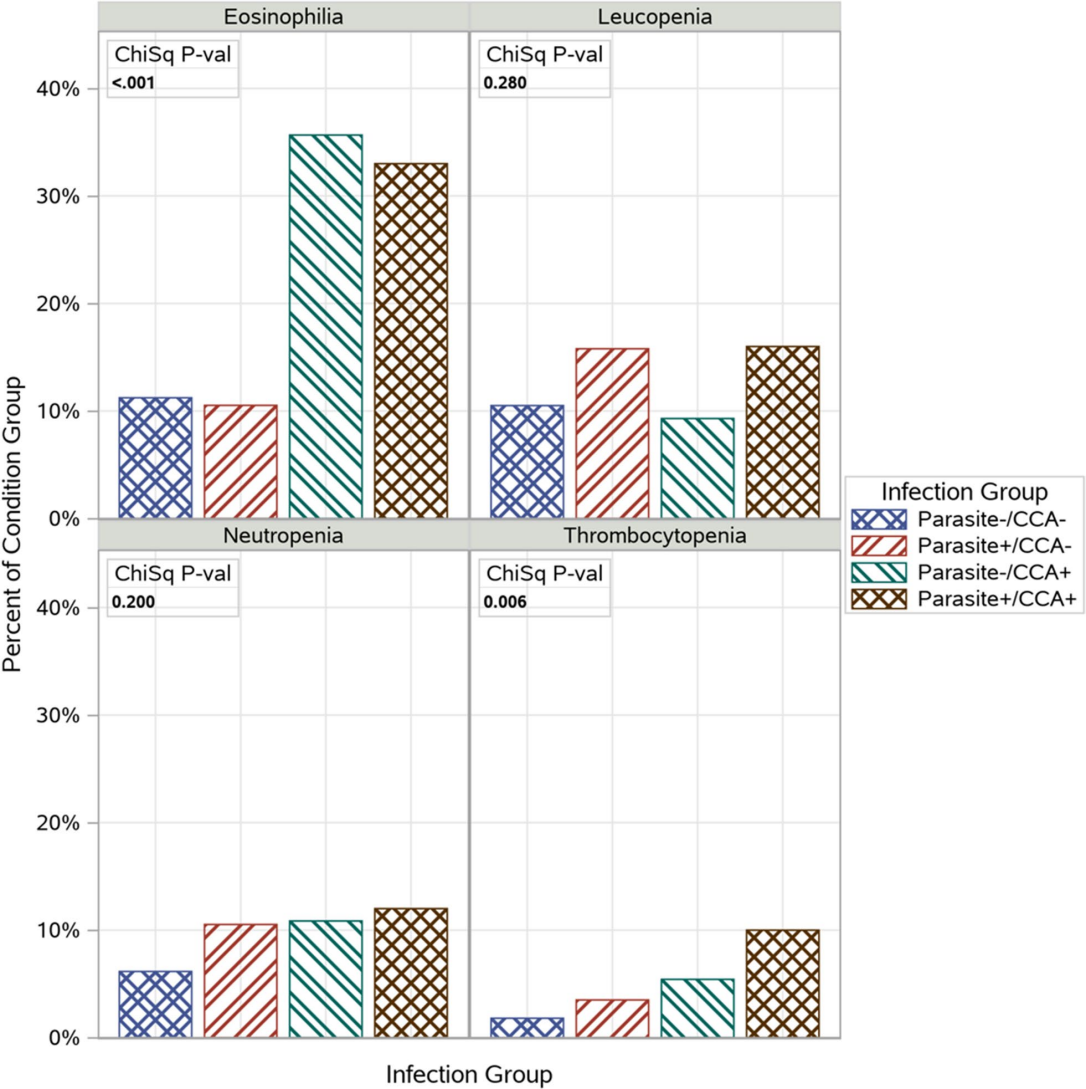
Prevalence of hematological clinical conditions based on infection group. Figure depicts the prevalence of selected hematologic conditions by the 4 infection groups. Across groups positivity was: Eosinophilia n = 116, Leucopenia n = 66, Neutropenia n = 49, and Leucopenia n = 24. P values reflect statistical independence between the hematologic condition and infection group categories

In order to assess the clinical significance of differences among hematology parameters in the four parasitemia/CCA infection group categories continuous, platelet, neutrophil, eosinophil and WBC counts were dichotomized into thrombocytopenia, neutropenia, eosinophilia and leucopenia clinical hematological conditions based on established cut-off ranges as indicated in the methods section. Eosinophilia was the most prevalent (20.6%) condition, whereas thrombocytopenia was the least prevalent (4.3%); the prevalence of neutropenia and leucopenia were 8.7% and 11.7%, respectively. We then performed a chi-squared test for independence between participant distribution in parasitemia/CCA infection group categories within each clinical hematological condition (Fig. 1). The univariate model revealed there were significant differences among the four parasitemia/CCA infection group categories for thrombocytopenia and eosinophilia (p = 0.006 and p < 0.0001, respectively), but not for neutropenia or leucopenia. The uninfected group category had the lowest proportion of individuals with thrombocytopenia and neutropenia followed by malaria parasitemia/schistosomiasis, and the highest proportion was among the co- infected group category. Schistosomiasis was associated with higher proportions of individuals with eosinophilia, whereas parasitemia was associated with modestly higher proportions of individuals with leucopenia.

**Fig. 2:**
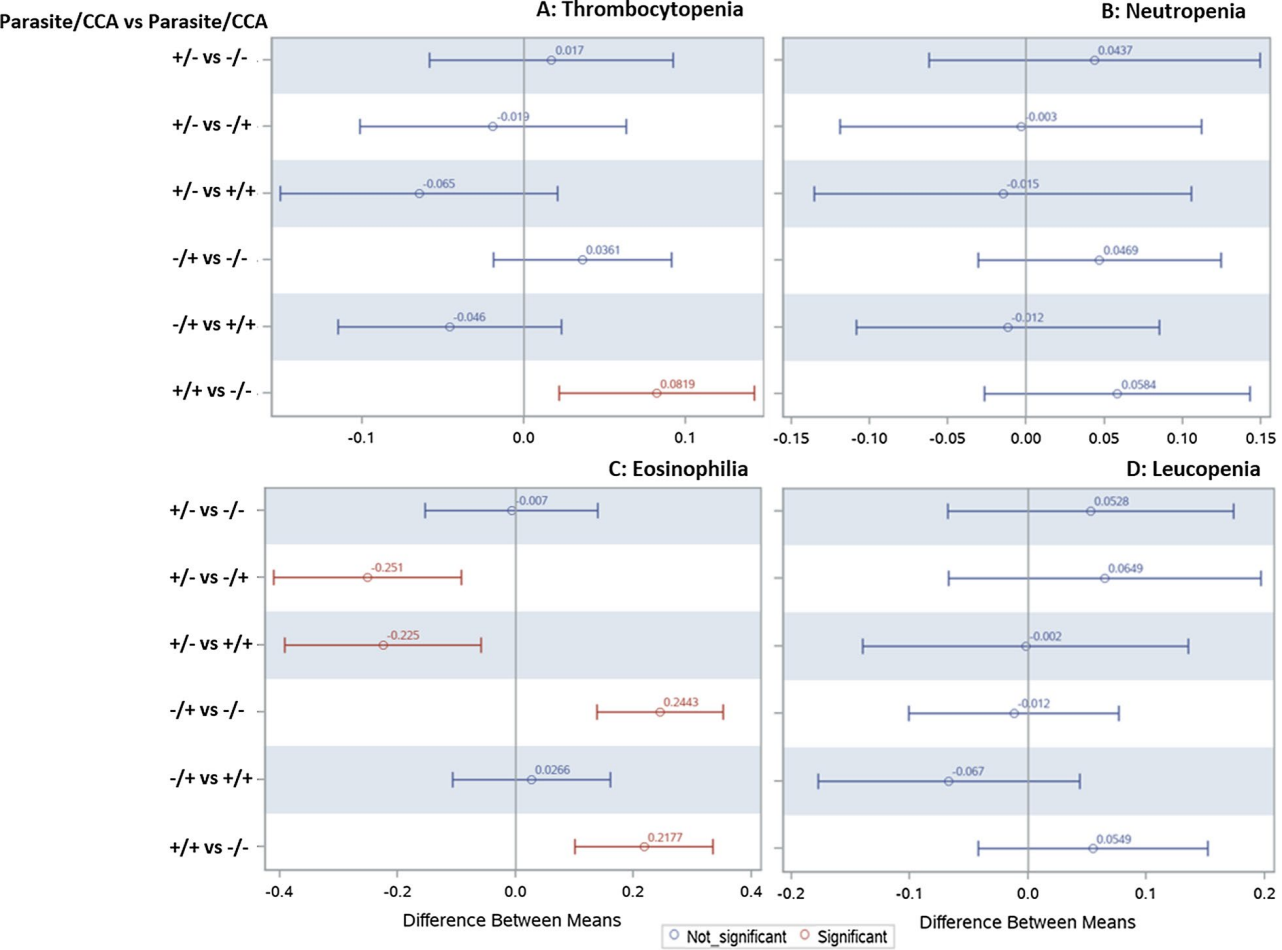
Categorical pairwise comparison for hematological clinical conditions based on infection group. Figure shows data generated using the generalized linear regression model for pairwise difference for **A** Thrombocytopenia, **B** Neutropenia, **C** Eosinophilia, and **D** Leucopenia. Pairwise differences in means which do not contain zero (shown in red) are significant

**Fig. 3:**
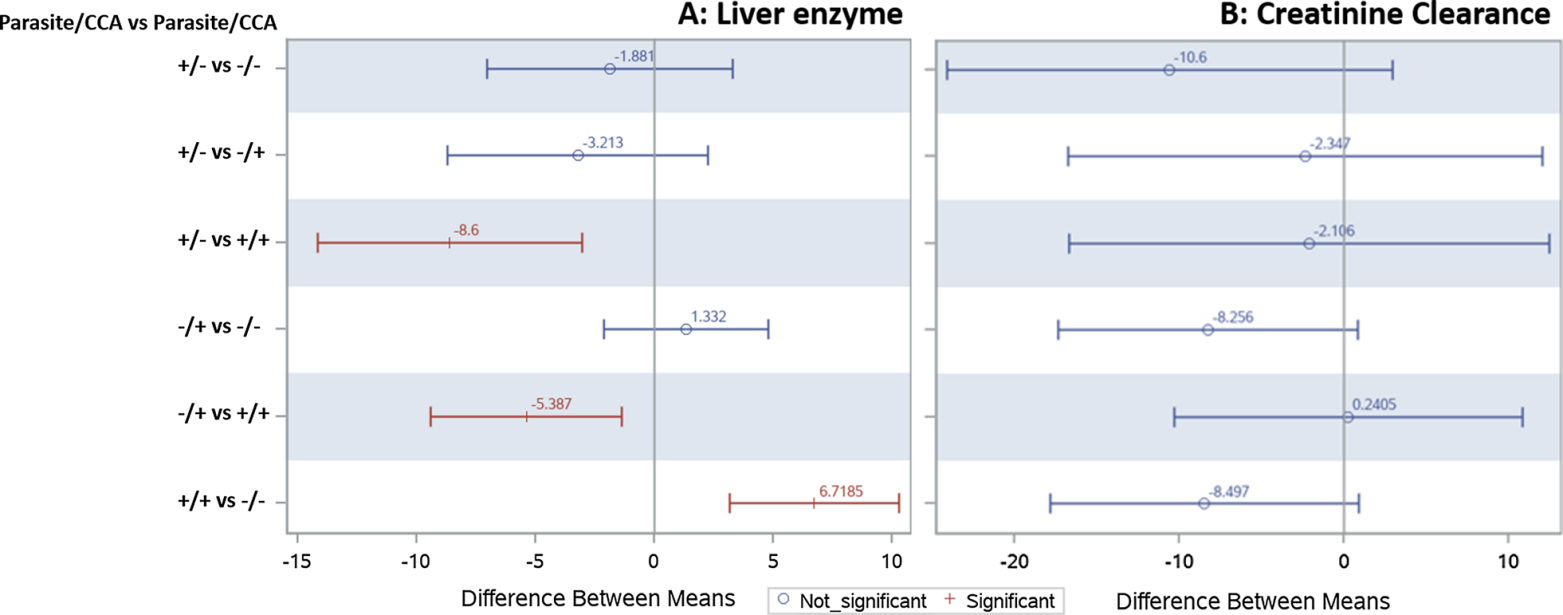
Categorical pairwise comparison for liver enzyme and kidney function based on infection group. Figure showing data generated using the generalized linear regression model for pairwise difference for **A** Liver enzyme, and **B** Creatinine clearance. Pairwise differences in means which do not contain zero (shown in red) are significant

To further interrogate the difference between the four infection group categories, we performed categorical pairwise comparison for the clinical hematological conditions (Fig. 2). The generalized linear regression model for pairwise difference showed confidence level of mean difference only in thrombocytopenia and eosinophilia (Fig. 2A, C). For thrombocytopenia, positive mean difference was only evident when the co-infected group category was compared to the uninfected. For eosinophilia, only groups with schistosomiasis infection (CCA +) showed a significant mean difference from other infection groups, suggesting that infection with schistosomiasis is associated with eosinophilia development, and that an association between *Plasmodium* infection and eosinophilia may be spurious. We then performed categorical pairwise comparison for blood chemistry (ALT and creatinine) among the four group categories; a significant mean difference between groups was evident only for ALT (Fig. 3). Pairwise comparisons between the infection groups for ALT level indicate that *Plasmodium* and schistosomiasis positive co-infection yielded significantly different enzyme levels from all other infection groups.

We then performed multivariate analysis using Poisson model with robust/sandwich estimator with independent variables of the clinical hematological groups and parasitemia/CCA status, gender, and age as independent predictors (Table 5). Multivariable models revealed statistically significant differences between infection groups for thrombocytopenia and eosinophilia, after adjusting for participant gender and age. For thrombocytopenia, parasitemia + /CCA + infection had a prevalence ratio of 4.04 compared to parasitemia−/CCA− group (p = 0.01), after adjusting for gender and age. An examination of eosinophilia indicated that prevalence of eosinophilia was significantly higher in the parasitemia−/CCA + and parasitemia + /CCA + groups than it was in the parasitemia−/CCA− infection group (PR 3.11 and 2.81, p < 0.0001, respectively). Additionally, female participants presented with lower prevalence of neutropenia (PR = 0.43, p = 0.01) and leucopenia (PR 0.31, p < 0.0001), compared to male participants after adjusting for age and infection status.

**Table 5:** Multivariate analysis using Poisson model with robust/sandwich estimator

	Thrombocytopenia PR (95% CI)	Neutrophilia PR (95% CI)	Eosinophilia PR (95% CI)	Leucopenia PR (95% CI)
Intercept	0.03 (0.01, 0.07)	0.10 (0.06, 0.18)	0.13 (0.09, 0.19)	0.18 (0.12, 0.27)
Parasite/CAA				
Parasite−/CCA−	Reference	Reference	Reference	Reference
Parasite + /CCA−	1.81 (0.37, 8.81)	1.57 (0.62, 3.93)	0.91 (0.40, 2.09)	1.36 (0.67, 2.76)
Parasite−/CCA +	2.66 (0.89, 7.94)	1.57 (0.80, 3.07)	**3.11** (2.08, 4.65)	0.76 (0.40, 1.42)
Parasite + /CCA +	**4.04** (1.37, 11.88)	1.48 (0.70, 3.11)	**2.81** (1.78, 4.44)	1.02 (0.56, 1.86)
Gender				
Male	Reference	Reference	Reference	Reference
Female	0.43 (0.16, 1.19)	**0.43** (0.21, 0.84)	0.86 (0.60, 1.23)	**0.31** (0.17, 0.56)
Age (years)				
18–25	Reference	Reference	Reference	Reference
26 +	0.96 (0.41, 2.25)	0.72 (0.39, 1.34)	0.82 (0.59, 1.15)	0.91 (0.54, 1.51)

*PR*  prevalence ratio

## Discussion

This study reveals a large proportion of our participants were infected with malaria (28.2%) or schistosomiasis (41.2%), and 18% were co-infected. Infection with either parasites was associated with changes in hematological parameters, liver enzymes and kidney function. Being infected with either malaria or schistosomiasis, or co-infection was not associated with anemia. Our univariate analysis indicated that thrombocytopenia and eosinophilia were significantly higher in co-infected participants than non-coinfected participants, and an association was not observed for neutropenia or leucopenia. Thrombocytopenia was more prevalent among the malaria and schistosomiasis co-infection group than other infection groups, and statistically different from those uninfected with either disease. The increased prevalence of thrombocytopenia was further corroborated through multivariate analysis, which suggested that even after controlling for age and gender, the prevalence of thrombocytopenia was significantly higher among the co-infected compared to the non-infected. Notably, multivariate analysis revealed the prevalence of neutropenia and leucopenia (independently) was significantly higher in men compared to women, which was not apparent when categorized based on infection status.

Our data underscore the importance of evaluating for co-infections in high endemic settings. For instance, initial analysis of malarial infection groups indicated significant differences in platelet count, percent monocytes, and eosinophils. Further, ALT values were significantly elevated (Table 2). However, when we separated data in the malarial parasite infected individuals from those infected with schistosomiasis and performed categorical analysis, malaria infection was only associated with significant group differences in platelet count (and not monocytes, eosinophils or ALT; Table 4 where we compared the first two columns [parasite−/CCA− vs parasite + /CCA−]), corroborating previous findings [10]. Co-infection was associated with dose-dependent hematologic responses that were significant for platelet count and percent neutrophils; platelets counts lowered to clinical thrombocytopenic levels but neutrophil levels did not reach clinical neutropenia.

Neutrophils play an important role in pathogen clearance by phagocytosis, and the activation and regulation of immune response [31, 32]. In children and immune-competent adults, neutrophil counts have been shown to increase in naturally-infected individuals during acute uncomplicated malaria, with the amount of neutrophil produced positively associated with parasitemia and disease severity [33, 34]. In non-immune travelers, neutrophil counts have been shown to increase with infection but do not vary with disease severity [33]. However, in asymptomatic *P. falciparum* infection, neutrophil numbers have been shown not to change [35] or decrease in *P. falciparum* infected pregnant women [36]. In our study, schistosomiasis infections (or co-infection with malaria) were not associated with neutropenia and leucopenia, corroborating previous studies [12, 37]. Many factors which may explain a gendered association with neutropenia/leucopenia were not included in the study as they are broadly out of scope.

Anemia and thrombocytopenia are the two hallmark hematological abnormalities associated with malarial disease. In our study of adult participants, there were no indications of an association between anemia and asymptomatic malaria infection or schistosomiasis parasites; there was an observed association with lower platelet count in malaria infected groups, but only reached clinical significance with schistosomiasis infection (Table 4). When we evaluated hematological parameters based on infection with malarial parasite only, platelet count reduced based on submicroscopic vs microscopic parasites. These findings demonstrate malaria infection in asymptomatic adults leads to reduction in platelet count.

Our results suggest this adult population may act as an important reservoir for *P. falciparum* and *Schistosoma* infections given the prevalence of asymptomatic infection. In recent years, considerable efforts and resources have been allocated in the control and elimination agenda of these two infections using various tools [1, 4]. Distribution of insecticide treated bednets and delivery of mass drug administration to high risk areas and populations are some of the strategies used in control and elimination efforts, but has mostly focused on children since they are the most vulnerable and are likely to develop overt symptoms [7, 22]. However, our study demonstrates that asymptomatic parasitic infections impact the health of adults, who may further act as reservoirs for transmission. Treatment of the asymptomatic adult population may serve an important role in reducing transmission, a crucial strategy towards control and elimination agenda for either of these diseases in endemic regions.

This study had several limitations. First, diagnosis for the presence of *P. falciparum* was based on molecular (nucleic acid detection) method whereas schistosomiasis was based on antigenic (protein/peptide) testing. Nucleic acid testing detects the presence of live parasites whereas antigenic testing may detect live or past infections, which may have led to overestimation of co-infections. The Schistosomiasis POC-CCA test has been shown to have high sensitivity for *S. mansoni* infections but low sensitivity for *S. haematobium* [48]. This may have led to underestimation of the schistosomiasis prevalence, which may have impacted the statistical analysis. However, in our region, *S. mansoni* is the most prevalent schistosomiasis and because of its expediency, POC-CCA test has continued to be used with satisfactory results even for the detection of *S. haematobium* (22, 49). Further, we did not quantify parasite infections by PCR, we only differentiated parasite density based whether parasites were microscopic (smear) vs submicroscopic (PCR). We also did not test the study participant for other infections including bacterial or other parasitic infections, which may skew the data. However, assuming that such undiagnosed infections would be equally distributed in the study groups, we believe our analysis provided reliable findings.

## Conclusion

Our data suggest that co-infection with *P. falciparum* and schistosomiasis may be associated with abnormal changes of hematological and blood chemistry parameters in adults living in high endemic regions of western Kenya, even when they present as asymptomatic infections. The observed hematologic effects appeared cumulative in those co-infected, which may indicate a not-inconsequential burden of disease in the population even in asymptomatic individuals, rather than existing as benign or trivial.

## Supplementary Information

**Additional file 1: Table S1.** Pairwise comparisons of Mean difference. **Table S2.** Pairwise comparisons of Mean difference. **Table S3.** Pairwise comparisons of Mean difference.

## Data Availability

The dataset supporting the conclusions of this article is available in the Harvard Dataverse repository, https://doi.org/10.7910/DVN/6A6XTZ,

## Abbreviations

ALT: Alanine aminotransferase
BMI: Body mass index
CCA: Circulating cathodic antigen
CrCL: Creatinine clearance
DSCF: Dwass, Steel Critchlow-Flinger method
IQR: Interquartile range
MCV: Mean corpuscular volume
PCR: Polymerase chain reaction
POC: Point-of-care
PR: Prevalence ratio
SGPT: Serum glutamate pyruvate transaminase
sSA: Sub-Saharan Africa
WBC: White blood count

## Declarations

### Disclaimer

Material has been reviewed by the Walter Reed Army Institute of Research. There is no objection to its presentation and/or publication. The opinions or assertions contained herein are the private views of the author, and are not to be construed as official, or as reflecting true views of the Department of the Army or the Department of Defense. The investigators have adhered to the policies for protection of human subjects as prescribed in AR 70–25.
