# Supplementary material for: Epidemiological and clinical implications of asymptomatic malaria and schistosomiasis co-infections in a rural community in western Kenya
Source: BMC Infect Dis. 2021 Sep 9;21:937. doi: 10.1186/s12879-021-06626-2 (PMC8431856; doi:10.1186/s12879-021-06626-2)
Supplement: Supplementary file 1 — Additional file 1: Table S1. Pairwise comparisons of Mean difference. Table S2. Pairwise comparisons of Mean difference. Table S3. Pairwise comparisons of Mean difference. [file 12879_2021_6626_MOESM1_ESM.docx]

Additional Tables

Manuscript: *Epidemiological and clinical implications of asymptomatic malaria and schistosomiasis co-infections in a rural community in western Kenya*

**Table S1: Pairwise comparisons of Mean difference**

|  |  | | | | | |
| --- | --- | --- | --- | --- | --- | --- |
|  | Thrombocytopenia | | | Neutropenia | | |
|  | Mean Diff | LCL | UCL | Mean Diff | LCL | UCL |
| cca-/par+ vs cca+/par- | -0.019 | -0.101 | 0.063 | -0.003 | -0.119 | 0.112 |
| cca-/par+ vs cca+/par+ | -0.065 | -0.151 | 0.021 | -0.015 | -0.135 | 0.106 |
| cca-/par+ vs cca-/par- | 0.017 | -0.058 | 0.092 | 0.044 | -0.062 | 0.149 |
| cca+/par- vs cca+/par+ | -0.046 | -0.115 | 0.023 | -0.011 | -0.108 | 0.085 |
| cca+/par- vs cca-/par- | 0.036 | -0.019 | 0.091 | 0.047 | -0.031 | 0.124 |
| cca+/par+ vs cca-/par- | 0.082 | 0.022 | 0.142 | 0.058 | -0.026 | 0.143 |
| par indicates parasitemia; CCA indicates schistosomiasis; LCL = lower confidence limit; UCL = upper confidence limit | | | | | | |
| **Table S2: Pairwise comparisons of Mean difference** | | | | | | |
|  | Table S2.2: Pairwise comparisons of Mean difference | | | | | |
|  | Eosinophilia | | | Leucopenia | | |
|  | Mean Diff | LCL | UCL | Mean Diff | LCL | UCL |
| cca-/par+ vs cca+/par- | 0.261 | 0.103 | 0.419 | 0.065 | -0.067 | 0.197 |
| cca-/par+ vs cca+/par+ | 0.242 | 0.078 | 0.407 | -0.002 | -0.140 | 0.136 |
| cca-/par+ vs cca-/par- | 0.021 | -0.123 | 0.165 | 0.053 | -0.068 | 0.174 |
| cca+/par- vs cca+/par+ | -0.019 | -0.151 | 0.113 | -0.067 | -0.178 | 0.044 |
| cca+/par- vs cca-/par- | -0.240 | -0.346 | -0.134 | -0.012 | -0.101 | 0.076 |
| cca+/par+ vs cca-/par- | -0.221 | -0.337 | -0.106 | 0.055 | -0.042 | 0.152 |
| par indicates parasitemia; CCA indicates schistosomiasis; LCL = lower confidence limit; UCL = upper confidence limit | | | | | | |
| **Table S3: Pairwise comparisons of Mean difference** | | | | | | |
|  |  |  |  |  |  |  |
|  |  | | | | | |
|  | Creatinine Clearance | | | ALT | | |
|  | Mean Diff | LCL | UCL | Mean Diff | LCL | UCL |
| cca-/par+ vs cca+/par- | -3.21 | -8.70 | 2.27 | -0.03 | -0.19 | 0.14 |
| cca-/par+ vs cca+/par+ | -8.60 | -14.15 | -3.05 | -0.02 | -0.19 | 0.14 |
| cca-/par+ vs cca-/par- | -1.88 | -7.04 | 3.27 | -0.12 | -0.27 | 0.03 |
| cca+/par- vs cca+/par+ | -5.39 | -9.42 | -1.36 | 0.0027 | -0.12 | 0.12 |
| cca+/par- vs cca-/par- | 1.33 | -2.13 | 4.79 | -0.09 | -0.20 | 0.01 |
| cca+/par+ vs cca-/par- | 6.72 | 3.15 | 10.29 | -0.10 | -0.20 | 0.01 |

par indicates parasitemia; CCA indicates schistosomiasis; LCL = lower confidence limit; UCL = upper confidence limit; ALT= alanine aminotransferase
